# Inguinal Endometriosis: An Uncommon Differential Diagnosis as an Inguinal Tumour

**DOI:** 10.5402/2011/272159

**Published:** 2010-11-04

**Authors:** Wu Shun Felix Wong, Chi Eung Danforn Lim, Xiping Luo

**Affiliations:** ^1^School of Women & Childrens Health, Faculty of Medicine, University of New South Wales, Sydney NSW 2052, Australia; ^2^South Western Sydney Clinical School, Faculty of Medicine, University of New South Wales, NSW 2052, Australia; ^3^Department of Gynaecology, GuangDong Hospital for Women and Children, Guangzhou Medical University, GuangDong 510182, China

## Abstract

Inguinal endometriosis can present as a rare tumor when it occurs outside the abdomen and pelvis. We present a patient with a painful tumor in the right inguinal region, where its diagnosis was made before the operation. This enabled a conservative excisional surgery to be performed. The literature is reviewed and the diagnosis as well as management are discussed.

## 1. Case History

A 48-year-old lady, gravida 3, para 3, presented with right inguinal mass associated with menometrorrhagia for eight weeks. Previously, she had regular monthly periods and no dysmenorrhea. She had a history of period pain at the groin during menses. On examination, the right inguinal mass measured 4 × 5 cm and was tender and relatively fixed. The cervix was normal and the uterus was bulky, mobile, and nontender. Pelvic ultrasound was normal, apart from a slightly bulky uterus. Endometrial sampling revealed proliferative endometrium. There was no evidence of polyp, hyperplasia nor malignancy. Fine-needle aspiration (FNA) biopsy of the inguinal mass was performed and revealed endometriosis. 

**Figure 1:**
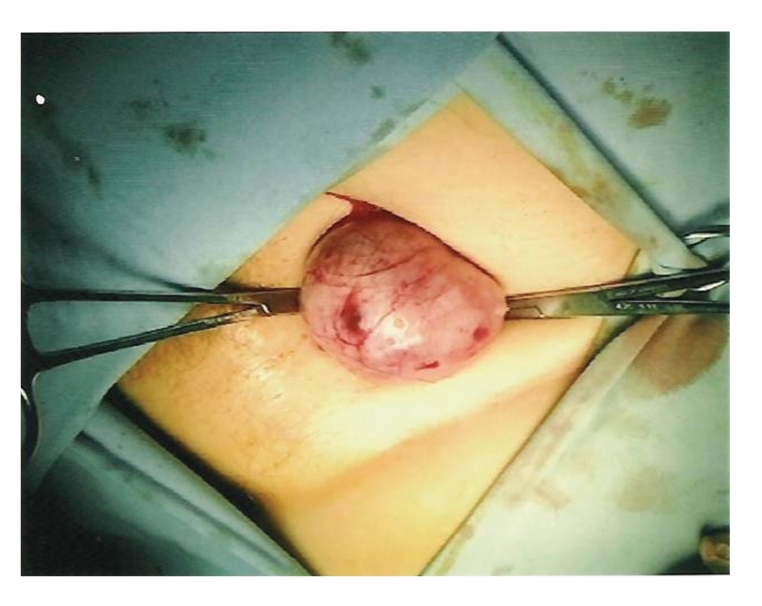
Inguinal mass, right.

**Figure 2:**
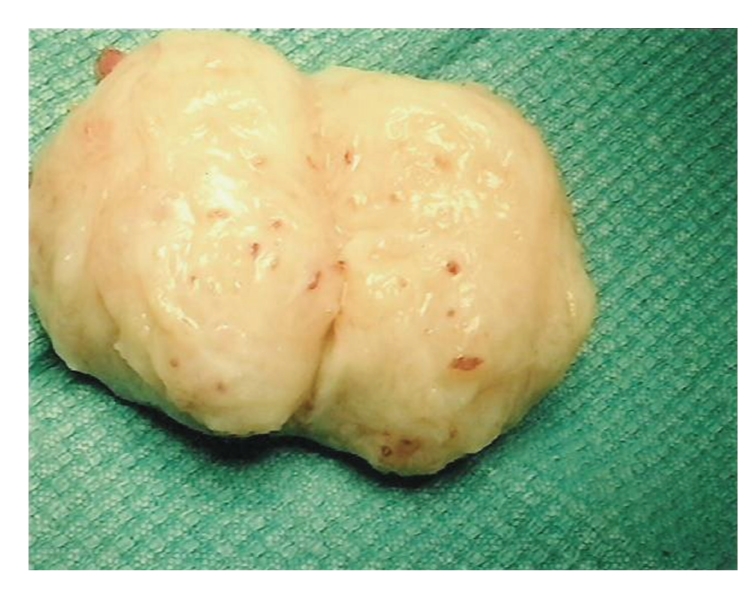
Gross specimen, right inguinal mass.

In view of her vaginal bleeding, bulky uterus, and inguinal mass, the patient was advised to have a dilatation and curettage along with excision of the groin mass. A 6 × 5 cm solid, fibroid-like tumor was removed from the right groin (see Figures 1 and 2). Histopathology study of the tumor confirmed deposits of endometrial type gland and stroma. The uterine curettage revealed disordered proliferating endometrium. 

## 2. Comments

Endometriosis is defined as the presence of endometrial glands and stroma outside the endometrial cavity and uterine musculature. Most common sites of endometriosis are within the pelvis, while unusual locations include bladder, intestine, surgical scars, diaphragm [1], umbilicus [2], and groin [3, 4]. Patients presenting with endometriosis of the inguinal region are few and far between. More specifically, the lesions can be found in the extraperitoneal portion of the round ligament [5], in the inguinal lymph nodes, in the subcutaneous adipose tissue, and even in the wall of sacs of inguinal or femoral hernias [6–9]. This paper aims to elucidate the incidence, pathogenesis, and management of this rare clinical problem. 

Inguinal endometriosis was first reported by Allen in 1896, but since then only a little more than 30 cases have been described [3, 6, 10, 11]. Battista Candiani et al. [11] reported six cases of inguinal endometriosis and they noted the mean age at diagnosis was 31 years [11]. The size of the mass reported ranges from 1 to 6 cm in diameter [8–11].  Table 1 summarized the reported cases in the literature. About 90% of reported cases of extraperitoneal endometriosis occur in the right inguinal area [4, 6, 10, 11] as was noted on the patient in this case.

Malignant transformation to carcinoma had been reported, with three cases of clear cell carcinoma documented [12]. Thus, it is of significance to remove the inguinal mass for pathology confirmation. 

The actual incidence of inguinal endometriosis is difficult to ascertain [11]. Due to the nonpelvic location of the lesion, patients would most often consult a general surgeon rather than a gynecologist, with a preoperative diagnosis of inguinal pathology. This could probably lead to underreporting of these cases. 

Battista Candiani et al. [11] describe six patients with inguinal endometriosis all whom have pelvic endometriosis on laparoscopy. They reported that 91% of inguinal endometriosis cases are associated with coexisting pelvic endometriosis [11]. Yet Inguinal endometriosis may be present without associated pelvic endometriosis [4]. Quagliarello et al. [8] reported a patient with inguinal endometriosis with no evidence of pelvic endometriosis on laparoscopy [8]. Laparoscopy was not performed on this patient because she was asymptomatic with no dysmenorrhoea or pelvic pain suggestive of the presence of pelvic pathology.

Although the typical complaints of patients with pelvic endometriosis include dysmenorrhoea, menstrual irregularities, dyspareunia, and infertility, patients with extrapelvic endometriosis like inguinal endometriosis present with unusual symptoms and occasionally, a diagnostic dilemma [6]. Periodic menstrual pain at the mass is sometimes the primary complaint [3, 10, 11]. Patients often present with a painful lump in the groin and premenstrual tenderness and/or swelling [6, 9, 10]. A history of a previous gynecologic procedure or surgical trauma is also contributory [3, 6]. Some patients may have no history of dysmenorrhoea, pelvic pain, or dyspareunia [3] as in our patient. However, she experienced right inguinal pain during menses.

On surgical exploration, a distinct firm mass was encountered [3]. Perez-Seoane et al. [7] described the gross findings of an inguinal endometrioma removed during surgery as multilobulated dark brown-colored tissue with hard consistency. Similar findings were earlier reported by Quagliarello et al. [8] in 1985. The histological diagnosis of endometriosis was based on the finding of characteristic endometrial glands with stromal cells within the fibrous connective tissue [3, 8, 9, 11] as was seen in this case. There was also chronic inflammatory cells [7] and hemosiderin-laden histiocytes [7, 11] found in some sections. 

Inguinal endometriosis may develop in the inguinal region as a mass from direct implantation, coelomic metaplasia, tubal regurgitation lymphatic spread [6]. However, Battista Candiani et al. [11] tend to dispute the plausibility of the latter two theories. They contest that, in view of the extraperitoneal location of the endometriotic lesions, tubal regurgitation seems unlikely. Furthermore, in terms of coelomic metaplasia, there does not appear to be an adequate embryologic explanation for the disproportionate predominance of endometriotic lesions on the right inguinal region, as compared to the left [11].

These theories do not offer an adequate explanation for the pathogenesis of inguinal endometriosis, especially in terms of patients with neither history of pelvic endometriosis nor previous surgery. According to separate accounts by Quagliarello et al. [8] and Seydel et al. [3], inguinal endometriosis is difficult to explain if it is not found in the pelvic lymph nodes [3, 8]. In separate reports, several authors have documented cases of inguinal endometriosis without evidence of concomitant pelvic endometriosis [7–10]. The lesions were localized either in the round ligament [10] or the hernial sac [7–9]. The mode of spread of endometriosis in these cases are still in question. 

The predominance of right-sided inguinal endometriosis remains unknown. The asymmetrical lymphatic drainage in favor of the right inguinocrural region might explain this phenomenon but according to Battista Candiani et al. [11], anatomical descriptions and oncologic observations do not seem to support this hypothesis [11]. 

Endometriosis of the inguinal canal can be difficult to detect, with the correct preoperative diagnosis made less than 50% of the time [3]. The presumptive diagnosis is most often confused with conditions such as incarcerated hernia, lymphadenopathy, suture granuloma, neuroma, abscess, hydrocoele of the inguinal canal, primary or metastatic cancer, lymphoma, lipoma, hematoma, sarcoma, and subcutaneous cyst [3, 10]. Very often, the final diagnosis is made only after the pathology, either from biopsy or at exploration, and demonstrates the histological presence of endometriosis [3]. 

A history of periodic menstrual pain and tenderness associated with an inguinal mass is important in distinguishing this condition from other inguinal pathology [10].

Diagnostic modalities such as radiological studies, ultrasound, and computed tomography scan have not been specifically helpful in the diagnosis [3]. These modalities cannot differentiate an endometrioma from a mass of similar density such as hematoma, lymphoma, sarcoma, or desmoid tumor [6]. On the other hand, magnetic resonance imaging is more accurate since it can identify the presence of iron in the haemosiderin deposits contained in an endometrioma [6]. Battista Candiani et al. [11] advocate more intensive use of this technique to increase the diagnostic accuracy [11]. Fine needle aspiration biopsy has also been shown to aid in the diagnosis of a case of endometriosis associated with an inguinal hernia [6, 7]. Perez-Seoane et al. [7] believe that this procedure should probably be the first step in the diagnosis of inguinal endometriosis due to its ease in performing and accuracy in results [7]. The finding of small groups of nonatypical epithelial cells in an inflammatory and proteinaceous background in FNA biopsy was similar to those of endometriosis in other sites. The absence of atypia helps to differentiate inguinal endometriosis from metastatic carcinoma to the inguinal region [7]. 

## 3. Use of Hormonal Therapy

Recurrences are rare after adequate surgical treatment. Hormonal therapy has shown to be a useful adjunct in the management of these cases. Batistta Candiani et al. [11] reported that in one out of the six patients with inguinal endometriosis treated by surgical excision, the inguinal mass recurred 14 years after the initial surgery [11]. This patient underwent a second exploration which revealed a multinodular subinguinal endometriotic lesion with ill-defined borders mixed with scar tissue and infiltrating the thigh muscles. Excision was incomplete and patient was given Danazol 400 mg/day for the residual endometriosis [11]. In terms of the usage of prophylactic hormonal therapy for endometriosis, Quagliarello et al. [8] (1985) cautioned that, due to its side effects, suppressive therapy with Danazol should be reserved only for patients with laparoscopic evidence of coexisting pelvic endometriosis [8]. 

## 4. Need for Laparoscopy

Majeski [6] recommends performing pelvic laparoscopy for all patients who have endometriosis in the extraperitoneal part of the round ligament or in a scar [6]. This view is shared by other authors (Goh and Flynn [10] 1994; Batistta Candiani et al. [11] 1991) because of the association with pelvic endometriosis and subfertility [10, 11]. On the other hand, Seydel et al. [3] did not see the need to perform laparoscopy in patients who do not present with signs of pelvic endometriosis [3]. 

For the patient in this case, simple excision was performed. It was not deemed necessary to perform laparoscopy since this patient did not present with signs of pelvic endometriosis.

## 5. Summary

We present a case of a 48-year-old woman with right inguinal mass associated with inguinal pain during menses. Fine-needle aspiration biopsy of the mass revealed endometriosis. This was managed by simple excision. The patient has remained asymptomatic ever since. Simple excision is deemed as adequate management in this case. 

Inguinal endometriosis is a rare clinical condition. It affects reproductive age women, with or without concomitant pelvic endometriosis. It usually presents as painful/tender inguinal mass and is most often mistaken for incarcerated hernia and other inguinal lesions. A history of catamenial pain often helps to rule out other inguinal pathology. 

Clinicians should not underscore the importance of a carefully taken history and physical examination, an increased awareness of this disease entity and a high index of suspicion in coming up with a correct diagnosis. Failure to recognize this syndrome and treatment with adequate excision may predispose to symptomatic recurrence. On the other hand, an accurate preoperative diagnosis may spare both the patient and physician the undue stress of performing an unnecessary emergency operation. 

## Figures and Tables

**Table 1:** Review of the literature.

Author	No. of patients	History of surgery	History of Pelvic endometriosis	Laparoscopy	Results of laparoscopy	Site of lesion
Majeski [6]	1	(+) previous CS	(−)	Done	(−)	Scar tissue
		(+) previous hernia				
Seydel et al. [3]	2	(+) previous CS	(−)	Not done	N.A.	No mention
Singh et al. [4]	1	(−)	No mention	Not done	N.A.	Hernial sac
	1	(+) previous CS	No mention	Not done	N.A.	Scar tissue
Goh and Flynn [10]	1	No mention	(−)	Not done	N.A.	Round ligament
Battista Candiani et al. [11]	6	No mention	All (+)	Done	All (+)	Round ligament
Perez-Seoane et al. [7]	1	(−)	(−)	Not done	N.A.	Hernial sac
Quagliarello et al. [8]	1	No mention	(−)	Done	(−)	Hernial sac
Brzezinskiand Durst [9]	1	No mention	(−)	Not done	N.A.	Hernial sac

## References

[1] Redwine DB (2002). Diaphragmatic endometriosis: diagnosis, surgical management, and long-term results of treatment. *Fertility and Sterility*.

[2] Victory R, Diamond MP, Johns DA (2007). Villar’s nodule: a case report and systematic literature review of endometriosis externa of the umbilicus. *Journal of Minimally Invasive Gynecology*.

[3] Seydel AS, Sickel JZ, Warner ED, Sax HC (1996). Extrapelvic endometriosis: diagnosis and treatment. *American Journal of Surgery*.

[4] Singh KK, Lessells AM, Adam DJ (1995). Presentation of endometriosis of general surgeons: a 10-year experience. *British Journal of Surgery*.

[5] Jenkins S, Olive DL, Haney AF (1986). Endometriosis: pathogenetic implications of the anatomic distribution. *Obstetrics and Gynecology*.

[6] Majeski J (2001). Scar Endometriosis Manifested as a Recurrent Inguinal Hernia. *Southern Medical Journal*.

[7] Perez-Seoane C, Vargas J, De Agustin P (1991). Endometriosis in an inguinal crural hernia. Diagnosis by fine needle aspiration biopsy. *Acta Cytologica*.

[8] Quagliarello J, Coppa G, Bigelow B (1985). Isolated endometriosis in an inguinal hernia. *American Journal of Obstetrics and Gynecology*.

[9] Brzezinski A, Durst AL (1983). Endometriosis presenting as an inguinal hernia. *American Journal of Obstetrics and Gynecology*.

[10] Goh JTW, Flynn V (1994). Inguinal endometriosis. *Australian and New Zealand Journal of Obstetrics and Gynaecology*.

[11] Battista Candiani G, Vercellini P, Fedele L, Vendola N, Carinelli S, Scaglione V (1991). Inguinal endometriosis: pathogenetic and clinical implications. *Obstetrics and Gynecology*.

[12] Ahn GH, Scully RE (1991). Clear cell carcinoma of the inguinal region arising from endometriosis. *Cancer*.

